# Pulsed Electric Fields Reshape the Malting Barley Metabolome: Insights from UHPLC-HRMS/MS

**DOI:** 10.3390/molecules30193953

**Published:** 2025-10-01

**Authors:** Adam Behner, Nela Prusova, Marcel Karabin, Lukas Jelinek, Jana Hajslova, Milena Stranska

**Affiliations:** 1 Department of Food Analysis and Nutrition, University of Chemistry and Technology, Technicka 3, 166 28 Prague, Czech Republic; adam.behner@vscht.cz (A.B.); nela.prusova@vscht.cz (N.P.); hajslovj@vscht.cz (J.H.); 2 Department of Biotechnology, University of Chemistry and Technology, Prague, Technicka 5, 166 28 Prague, Czech Republic; marcel.karabin@vscht.cz (M.K.); lukas.jelinek@vscht.cz (L.J.)

**Keywords:** pulsed electric field (PEF), malting barley, UHPLC-HRMS/MS, metabolomics, biomarkers

## Abstract

The Pulsed Electric Field (PEF) technique represents a modern technology for treating and processing food and agricultural raw materials. The application of high-voltage electric pulses has been shown to modify macrostructure, improve extractability, and enhance the microbial safety of the treated matrix. In this study, we investigated metabolomic changes occurring during the individual technological steps of malting following PEF treatment. Methanolic extracts of technological intermediates of malting barley were analyzed using metabolomic fingerprinting performed with UHPLC-HRMS/MS. For data processing and interpretation, the freely available MS-DIAL—MS-CleanR—MS-Finder software platform was used. The metabolomes of the treated and untreated barley samples revealed significant changes. Tentatively identified PEF-related biomarkers included 1,2-diacylglycerol-3-phosphates, triacylglycerols, linoleic acids and their derivatives, octadecanoids, N-acylserotonins, and very long-chain fatty acids, and probably reflect abiotic stress response. Monitoring of the profiles of selected biomarkers in PEF malting batch indirectly revealed a potential enhancement of enzymatic activity after the PEF treatment. These results contribute to fundamental knowledge regarding the influence of PEF on final malt from a metabolomic perspective.

## 1. Introduction

The pulsed electric field (PEF) technique represents a highly promising and effective solution in various areas of the food processing industry [1]. This non-thermal method, based on the application of very short electrical pulses with defined parameters (pulse length, voltage, current, frequency, polarity, and others) has found applications such as enhancing extractability [2,3,4], improving protein digestibility [5,6], and serving as a non-thermal preservation treatment for many types of matrices [7]. During treatment, the electrical pulses pass through the matrix which must be capable of conducting an electric current [8], and induce changes at the ultrastructural level, affecting macromolecules [9,10,11] as well as smaller molecular structures [12,13,14].

The range of PEF treatment applications is currently very broad, including, for example, the processing of cereals [15]. Very interesting results have been observed regarding the application of PEF treatment to enhance seed germination [16]. Germination represents a fundamental technological step in malting, which is an integral part of the production of beer—one of the most popular and widely consumed beverages worldwide [17,18]. To initiate grain germination, the grains must be steeped first. That represents an ideal phase for implementing PEF treatment due to the technological presence of water as an electrolyte [19]. Saxton et al. [20] treated malting barley with the PEF technique and reported accelerated malt germination and improvements in the technological parameters of the resulting malt products. Similar improvements in the technological parameters of malt following PEF treatment were also observed by Zhang et al. [21] and Polachini et al. [22].

At the same time, favorable conditions such as temperature (14–18 °C) and high humidity (95–100% air humidity) during barley germination after steeping also promote the development of microorganisms and increase the occurrence of potential contaminants, posing a risk to consumers [23]. In this context, PEF treatment offers a promising solution due to its microbial decontamination capability [7]. During treatment, the electrical pulses disrupt the cell walls of pathogenic microorganisms and their spores, thereby deactivating and/or killing them [14,24]. The positive effect of PEF treatment has been observed with various microorganisms [7,25] including *Fusarium graminearum* in seeds of various crops [26]. A recent study by Behner et al. [24] described significant metabolomic changes, a decrease in viability, and reduced mycotoxin production by four highly pathogenic *Fusarium* species (*F. culmorum*, *F. graminearum*, *F. poae*, *F. sporotrichioides*) following PEF treatment. In the study by Karabín et al. [19] malting barley was treated with the PEF technique, resulting in reduced growth of *Fusarium* pathogens (*F. culmorum*, *F. graminearum*, *F. poae*, *F. sporotrichioides*) and a decrease in the presence of mycotoxins in the final malt. At the same time, it was demonstrated that PEF treatment did not negatively affect the germination capacity of the malt and the technological parameters remained satisfactory [19]. The direct effect of PEF treatment on the mycotoxin molecules in malting barley was investigated by Stránská et al. [14], who identified degradation and transformation products of selected mycotoxins (Enniatin A1 and B1, Tentoxin, Zearalenone) as a result of PEF-induced oxidative and hydrolytic reactions. These findings support the direct influence of PEF on the physicochemical characteristics of the entire matrix. However, to the best of our knowledge, a comprehensive evaluation of biological changes at the metabolomic level in malting barley following PEF treatment has not yet been published. The objective of this study was to describe the metabolomic changes occurring in malted barley throughout the entire malting process after PEF treatment. Furthermore, the metabolomic profiles of untreated malted barley during the malting process were of particular interest.

## 2. Results

### 2.1. Extraction Method Optimization and UHPLC-HRMS/MS Analysis

A fundamental step of the UHPLC-HRMS/MS fingerprinting method was the optimization of the extraction procedure. To ensure coverage of a wide range of metabolite polarities, five extraction solvents or solvent mixtures of varying composition were tested; the extraction systems included methanol:propan-2-ol (50:50, *v*/*v*), methanol, methanol/water (50:50, *v*/*v*), and water. Based on the results summarized in Table S1, methanol provided the best performance, yielding a total of 16,547 detected features (11,105 in ESI+ and 5442 in ESI−). Figures S1–S5 in the Supplementary Materials present the TIC (total ion current) chromatograms of the UHPLC-HRMS/MS fingerprints for the PEF-treated and control samples. At first glance, the TIC profiles of the PEF-treated and control samples (see Figures S1–S5) appear very similar; however, subsequent chemometric analysis revealed clear distinctions.

### 2.2. Data Processing Optimization

The optimization of a crucial processing parameter in the MS-DIAL (v. 4.80, 2021, RIKEN, Japan) software, the *Minimum peak height*, was also part of this study. In MS-DIAL, the *Minimum peak height* parameter, located within the *Peak detection* tab, specifies the lowest signal intensity threshold required for a peak to be classified as valid. Signals not meeting this criterion are discarded as background noise. The optimal setting for this parameter is contingent upon the characteristics of the analytical instrument and the dataset; however, in general, increasing the threshold mitigates noisy data but may inadvertently eliminate authentic signals of low abundance [27]. The setting of this parameter thus fundamentally affects the selection of variables that will subsequently undergo statistical analysis and, in turn, determine the statistically significant features representing the final biomarkers—unique diagnostic metabolites induced exclusively by the response of barley to PEF treatment. The optimization of the *Minimum peak height* parameter was conducted using a single dataset (dataset D, posing the richest metabolite profile), with the *Minimum peak height* levels of 0, 1000, 4000, 7000, and 10,000 chosen in accordance with the software recommendations (setting of 1000 up to 10,000 for QTOF systems). Dataset D was reprocessed for each *Minimum peak height* value following the procedure described in Section 4.7 and Section 4.8. It is worth noting that the quality of the raw LC-MS data for the selected statistically significant features had to be verified manually. This is because, during automatic software peak picking and data alignment, some features with insufficient data quality can pass the defined quality criteria. Similar findings have been reported previously [27]. Using SCIEX OS software (v. 1.5.0.23389, SCIEX, Concord, ON, Canada), extracted ion chromatograms (XICs) (fulfilling criteria mass detection error < 5 ppm) were manually created based on the exact *m*/*z* values generated by the MS-DIAL software, and peak shapes were evaluated. In cases of unsatisfactory peak shape quality, the respective “noisy data variables” were excluded. As shown in Figure S6, when the *Minimum peak height* parameter was set to 7000 or higher, no variables needed to be discarded due to poor LC-MS data quality. Therefore, the *Minimum peak height* parameter of 7000 was used to process the complete dataset.

### 2.3. Chemometric Analysis

**Figure 1:**
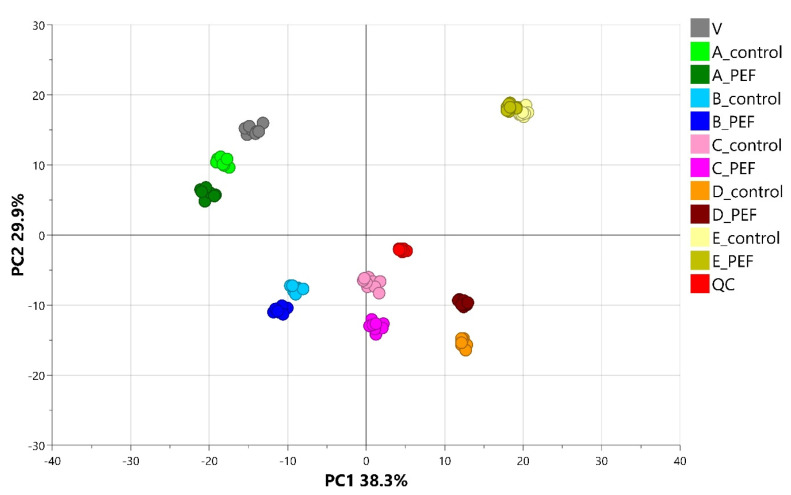
The PCA (Score scatter plot) including all samples (V—Input barely (0 h; n = 10), A—Pre-soaked barley (10 min; n = 10), B—Steeped barley (48 h; n = 10), C—Green malt I (72 h; n = 10), D—Green malt II (120 h; n = 10), E—Final barley malt (168 h; n = 10), QC—Quality control; n = 18) colored according to the particular experimental conditions.

The raw data files generated by the UHPLC-HRMS/MS analysis were processed according to the workflow described in Section 4.7. In the first step, all data were processed together, and the application of PCA provided an initial statistical overview of the differences between the metabolomes of the various sample groups. Figure 1 shows the PCA score scatter plot of the entire dataset, with samples colored according to the individual technological steps of malting and the PEF-treated or untreated control groups. The clusters of samples corresponding to the different malting phases are clearly distinguishable in the overall PCA score plot, indicating pronounced differences in the metabolomes associated with each phase. The sequence of the individual malting phases, as seen in the PCA score plot (Figure 1), aligns with the time progression of the malting process. Less distinct clusters for the PEF-treated and control samples are evident within each individual malting phase; nevertheless, substantial metabolic differences between PEF-treated and control samples at each stage of the malting process are still evident.

**Figure 2:**
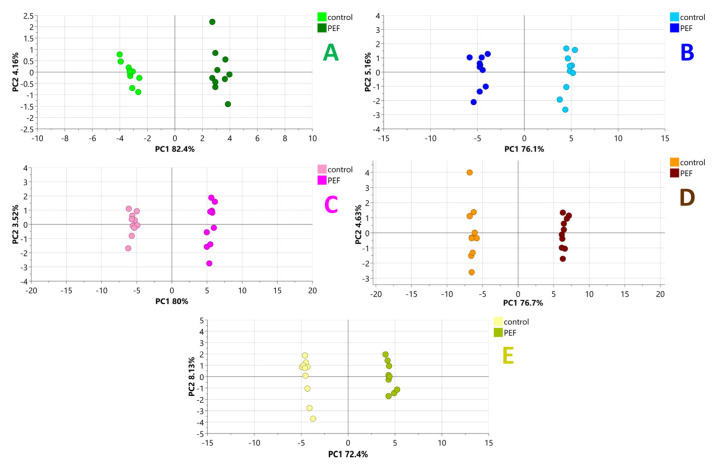
The PCA (Score scatter plots) of each individual technological steps of malting (**A**)—Pre-soaked barley (10 min; n = 10)**,** (**B**)—Steeped barley (48 h; n = 10), (**C**)—Green malt I (72 h; n = 10), (**D**)—Green malt II (120 h; n = 10), (**E**)—Final barley malt (168 h; n = 10)) colored according to the particular experimental conditions (PEF/control).

To emphasize the differences in the metabolomes of PEF-treated and control samples, the next step involved constructing binary models (*Horizontal* data processing approach, see Section 4.8). The subsequent PCA analyses were performed for each technological step, and as illustrated in Figure 2, the cluster separation improved, demonstrating promising potential for the development of supervised multivariate models and the identification of statistically significant biomarkers.

To identify the features most responsible for the distinct clustering of samples between the PEF-treated and control groups across all datasets, supervised OPLS-DA models were developed (see Figure S7). The quality parameters for all models are provided in Table S2. Model validation was conducted using seven-fold cross-validation. *R^2^* and *Q^2^* parameters reached values close to 1, which, according to the classification of Triba et al. [28], indicates an excellent description of the data and high predictive ability of the models. The key features driving the separation of PEF-treated and control samples, serving as biomarkers indicative of metabolic changes in barley following PEF treatment, were selected through Variable Importance of the Projection (VIP) plots (VIP score > 1) and Receiver Operating Characteristics (ROC) analysis (AUC = 1) simultaneously.

**Figure 3:**
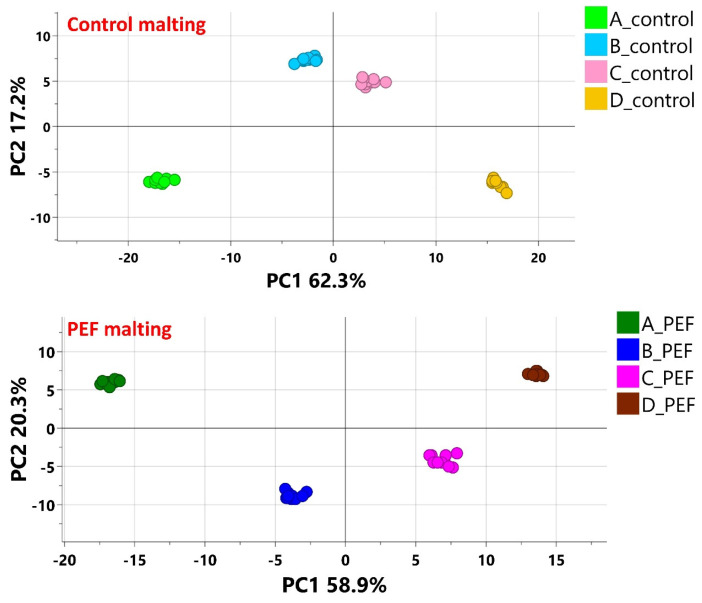
The PCA (Score scatter plots) of each batch of malting (PEF malting vs. control malting). All individual technological steps of malting (A—Pre-soaked barley (10 min; n = 10), B—Steeped barley (48 h; n = 10), C—Green malt I (72 h; n = 10), D—Green malt II (120 h; n = 10)) are clearly clustered in both types of malting experiments.

The *Vertical* data processing approach enabled the monitoring of profiles of selected markers in individual malting batches (PEF malting vs. control malting). Due to thermal interference during kilning, which affects the thermolabile fraction of the barley metabolome, the final malt samples (dataset E) were excluded from the *Vertical* approach. From Figure 3, which shows the PCA score plots, it is evident that well-separated clusters represent the individual malting phases, maintaining the same sequence for both the control and PEF malting experiments according to principal component 1 (PC1). Pattern Hunter (MetaboAnalyst) was used to select biomarkers exhibiting increasing or decreasing trends during both malting experiments.

### 2.4. PEF-Related Biomarkers and Their Ontologies

Through the *Horizontal* data processing approach, two categories of statistically reliable features were selected for each dataset: PEF-related features, indicating biomarkers that were up-regulated following PEF treatment, and control features, representing biomarkers that were down-regulated in response to PEF. Specifically, for pre-soaked barley (dataset A), 22 PEF-related biomarkers were identified (20 up-regulated, 2 down-regulated); for steeped barley (dataset B), 108 biomarkers (65 up-regulated, 43 down-regulated); for green malt I (dataset C), 69 biomarkers (58 up-regulated, 11 down-regulated); for green malt II (dataset D), 122 biomarkers (55 up-regulated, 67 down-regulated); and for final malt (dataset E), 50 biomarkers (24 up-regulated, 26 down-regulated). All of these biomarkers are detailed in the Supplementary file **Candidates of PEF-related biomarkers Horizontal**, which includes their mass spectrometric characteristics and VIP/ROC selection parameters. Among the extensive list of PEF-related biomarkers, chemical similarities were identified for individual markers. In addition to the proposed name of each biomarker candidate, the MS-FINDER software (v. 3.52, 2021, RIKEN, Japan) classified them into chemical groups (ontologies). The unique biomarker ontologies occurring the most frequently (two or more times) within each malting step are summarized in Table 1.

Data processing using the *Vertical* approach enabled the monitoring of the profiles and temporal evolution of selected biomarkers in individual malting batches (PEF malting vs. control malting). The Pattern Hunter algorithm identified a total of 83 biomarkers with an increasing trend (1-2-3-4) in the ‘A-B-C-D’ direction of malting process, and 31 biomarkers with a decreasing (4-3-2-1) trend in the ‘A-B-C-D’ direction of malting process for the PEF malting batch, as well as 60 biomarkers with an increasing pattern and 27 with a decreasing pattern for the control malting batch across the malting experiments. All of these biomarkers, along with their mass spectrometric features, are provided in the Supplementary file **Candidates of PEF-related biomarkers Vertical**. Similarly to the *Horizontal* approach, common chemical groups (ontologies) generated by MS-FINDER were also observed. The unique ontologies of biomarker candidates occurring most frequently (two or more times) for each malting batch (PEF malting and control malting), with either an increasing (1-2-3-4) or decreasing (4-3-2-1) trend in the ‘A-B-C-D’ direction of malting process, are summarized in Table 2. All unique ontologies of the selected biomarkers and their trends for each specific dataset group (PEF or control) are visualized in heatmaps (Figures S8 and S9 in the Supplementary Materials).

## 3. Discussion

In view of the persisting issue of contamination of major crops by micromycetes and the associated formation of mycotoxins, measures to prevent the growth of micromycetes and the production of mycotoxins are of critical importance. Favorable conditions (optimal temperature and humidity) during barley germination, a fundamental step in the malting process, promote the development of toxinogenic micromycetes and increase the occurrence of potential contaminants [23]. The pulsed electric field (PEF) technique represents a promising solution for their elimination. It can be assumed that the metabolic activity of grains is also impacted at the same time. To the best of our knowledge, the direct effect of PEF treatment on the metabolome of malting barley has not yet been described. The impact of PEF treatment on the malting process itself, particularly regarding the technological quality of the final malt, has been investigated in previous studies [19,20,22]. Saxton et al. [20] reported that PEF accelerated the germination rate of barley and improved both the quality and yield of malt extract. A recent study by Karabin et al. [19] demonstrated that under optimized conditions, PEF treatment does not negatively affect the quality of the final malt, and at the same time, decreases the levels of toxinogenic fungi, as well as mycotoxins. Our metabolomic fingerprinting results represented by the PCA (Figure 1) clearly illustrate the progression of individual malting phases for both PEF-treated and control samples. The order and overall pattern of the malting phases from input barley (V) to final malt (E) are comparable between the control and PEF-treated experiments. A similar order and pattern of the malting phases was also observed by Zhao et al. [29], who conducted a time-course comparative metabolomic analysis of different barley cultivars during malting, including cultivars specifically intended for malting.

However, when focusing on the individual malting phases depicted in Figure 1, the outcomes of metabolomic fingerprinting using advanced, highly sensitive high-resolution mass spectrometry reveal clear differences between PEF-treated samples and the control group. These differences become even more pronounced when the PCA score plots in Figure 2 are constructed following independent data processing of each malting phase. The observed differences in the metabolome can be attributed to the PEF treatment. The workflow based on the study by Behner et al. [24], and improved by optimization of the *Minimum peak height* processing parameter (see Section 2.2 Data Processing Optimization), enabled a more detailed identification and characterization of PEF-induced changes in the metabolome. This approach led to the identification of numerous statistically significant and robust PEF-related biomarkers that were either up- or down-regulated as a result of PEF treatment.

As concerns the unambiguous identification of metabolites (or metabolic pathways intermediates), ’non-model’ organisms like barley still pose a challenge for researchers because, unlike well-studied model organisms such as *Arabidopsis thaliana* [30,31,32], their metabolic pathways are not elucidated in metabolomic maps and pathway software. This limited knowledge complicates the interpretation of experimental results. Nevertheless, we attempted at least a partial biological interpretation of the selected biomarkers grouped within the unique ontologies listed in Table 1, with a particular focus on PEF-up-regulated biomarkers. The complete list of biomarkers identified is provided in detail in the Supplementary files **Candidates of PEF-related biomarkers Horizontal** and **Candidates of PEF-related biomarkers Vertical**, together with their mass spectrometric characteristics and statistical selection parameters.

1,2-diacylglycerol-3-phosphates represented one of the most frequently occurring ontologies in PEF-treated pre-soaked barley (dataset A). This ontology included biomarker *phosphatidic acid (18:1(9Z)/20:4(5Z*,*8Z*,*11Z*,*14Z))*, which belongs to the chemical group of phosphatidic acids (PAs). In general, PAs play a key role as important signal-transducing molecules in plants and are involved in processes associated with the plant’s response to environmental stresses [33,34].

For steeped barley (dataset B), the most frequently occurring ontology was linoleic acids and derivatives in the case of PEF-related up-regulation. This ontology was represented by *α-dimorphecolic acid* biomarker, which has been previously found in rice (*Oryza sativa*) plants as a metabolomic response and part of a self-defensive mechanism to blast disease infection caused by the pathogenic fungus *Magnaporthe oryzae* [35,36,37]. Another PEF-induced up-regulated metabolite from the linoleic acids and derivatives chemical group was *corchorifatty acid F (9*,*12*,*13-trihydroxy-10*,*15-octadecadienoic acid)*, which has been identified as an infection-response marker in *Arabidopsis thaliana* infected by *Vericillium longisporum* [38,39]. This compound also greatly reduced infection of the powdery mildew fungus *Blumeria graminis* after treatment of infected first leaves of barley (*Hordeum vulgare*) seedlings [40]. In steeped barley (dataset B), the green malt I (dataset C) and green malt II (dataset D) groups of triacylglycerol metabolites were the most commonly occurring ontologies up-regulated after PEF treatment. An increase in triacylglycerols was also observed by Navari-Izzo et al. [41] in cultivars of barley exposed to low levels of SO_2_ fumigation. This stress exposure affects lipid metabolism and functions with membrane stabilization, which could be comparable with PEF treatment exposure. Another observed ontology of octadecanoids was also up-regulated after PEF treatment in steeped barley (dataset B). One of the identified biomarkers, *12-oxo-phytodienoic acid* (OPDA), represents an intermediate in the *jasmonic acid* (JA) biosynthesis pathway contributing to plant defense against insect pests. OPDA stimulates enhanced resistance in rice and wheat (*Triticum aesetivum*) against brown planthopper [42] and hessian fly [43,44]. Accumulation of OPDA associated with higher resistance against *Bradysia impatiens* insect in plants from the *Arabidopsis* family was described by Stintzi et al. [45]. According to Maucher et al. [46], JA and its precursor—OPDA—play a key role in the adaptation of plants to abiotic stress, which, in our case, could be represented by PEF treatment. We assume that the up-regulation of these metabolites, which have been described in previous studies as markers of stress in response to plant pathogens, is directly linked to the abiotic stress caused by pulsed electric field (PEF) treatment.

In green malt I (dataset C), one of the most prevalent up-regulated ontologies was *N*-acylserotonins, represented by *feruloyl-serotonin* and *coumaroyl-serotonin*, which belong to the hydroxycinnamic acid amide chemical group. The up-regulation of *feruloyl-serotonin* and *coumaroyl-serotonin* metabolites has been previously described also in the context of plant responses to abiotic stress [47]. These compounds play crucial roles in plant protection under various extreme conditions [48]. We hypothesize that the up-regulation of these metabolites may result from the exposure of PEF-treated samples to electrical pulses. Elevated levels of these metabolites have also been observed in rice and potatoes as a response to infection or biological attack [49]. Although these metabolites are relatively widespread throughout the plant kingdom, their precise biological function remains poorly understood [47].

Up-regulation of very long-chain fatty acids (VLCFAs) was observed in green malt II (dataset D) and the final barley malt (dataset E). In green malt II, this ontology was specifically represented by the biomarkers *linearmycin A* and *melissic acid A (triacontanoic acid)*. *Linearmycin A* has been described as a secondary bacterial metabolite [50], tested for its potential in biotic stress management and plant disease control due to its antifungal properties [51]. *Melissic acid A (triacontanoic acid)* represents one of the major compounds found in the leaf cuticular waxes of *Ricinus communis* L. [52]. Cuticular waxes and their composition may play a crucial role in drought tolerance of economically important crops such as barley [53]. Additionally, cuticular waxes contribute to defenses against pathogens and UV damage [54,55]. In general, VLCFAs are involved in various plant responses to both biotic and abiotic stresses [56]. It is assumed that the up-regulation of VLCFAs in treated samples also reflects an adaptive response of malt to PEF treatment.

One of the identified biomarkers in green malt II (dataset D) and in the final barley malt (dataset E) was *hordatine B*. This biomarker was down-regulated following PEF treatment in both datasets (D and E). *Hordatine B* belongs to the group of hordatines, which represents unique metabolites from the phenolamide chemical family, typical of barley (*Hordeum vulgare L.*). The antifungal properties of these specialized metabolites have been demonstrated in numerous studies [47,57,58,59]. Their increase as a plant response to pathogen interaction has been well documented in barley seedlings [47]. In the context of PEF treatment, the down-regulation of this antifungal defense metabolite could be attributed to the possible suppression of the proliferation of pathogenic fungi in the PEF-treated grains. This hypothesis is supported by the findings of a study by Prusova et al. [60], which observed a significant reduction in pathogenic fungi of the genus *Fusarium* in PEF-treated malts, accompanied by a decrease in mycotoxins from the A trichothecene family.

In the *Vertical* analysis, attention was focused on markers showing an increasing trend (1-2-3-4) in the ‘A-B-C-D’ direction of malting, and on comparing the total number of these markers and the unique chemical groups present in the PEF and control malting experiments. In the PEF malting experiment, a higher overall number of markers with an increasing trend was observed, along with a greater number of unique chemical groups represented by two or more biomarkers (see Table 2 and Supplementary file **Candidates of PEF-related biomarkers Vertical**). For example, in PEF-treated samples, an increase was observed in metabolites from the xanthophyll group, specifically *carpoxanthin* and *15*,*15′-dihydroxy-beta-carotene*, which are responsible for the color of the grain and endosperm [61]. A further examination of the unique chemical groups and metabolites with an increasing trend (1-2-3-4) in PEF-treated samples suggests that PEF technology may support the germination of treated grains. Enhanced germination in PEF-treated barley seeds has been reported by Saxton et al. [20], Shi et al. [62], and Lynikiene et al. [63]. Increased activity of certain enzymes, such as those from the lipase group, is known to be associated with barley germination [64]. We assume that evidence of increased lipase activity in PEF-treated samples is reflected in the higher levels of metabolites from the linoleic acids and derivatives, fatty acid esters, and long-chain ceramides/fatty acids/fatty alcohols group (see Table 2). The rise in these lipid metabolites may therefore be indirectly linked to sprout formation, consistent with the observations previously reported by Al-Taher et al. [65]. During malting, an increase in the activity of proteolytic enzymes [66,67] was also indirectly observed, which in our case may be reflected by the increased levels of metabolites from the N-acyl-amine group in PEF-treated samples (see Table 2). A deeper understanding of the origin and integration of selected biomarkers into metabolomic pathways remains challenging, as the current knowledge of the secondary metabolome of a wide range of plants, including more than 200,000 metabolites, specifically *Hordeum vulgare* plants, has not yet been explored in depth [68,69]. While an exhaustive biochemical explanation is beyond the scope of the current study, the presented data lay an essential groundwork for subsequent research incorporating integrative “omics” approaches, such as transcriptomics and proteomics. The secondary metabolites linked to PEF treatment, as identified in this study, were generated solely through the application of PEF to malting barley grains. Importantly, these changes did not exert any adverse influence on the technological properties of the resulting malt, while in relation to mold growth and mycotoxin formation, PEF demonstrated predominantly beneficial effects in terms of their mitigation. Although metabolomic studies generally involve a broader range of biological material, such as different barley varieties or harvesting years, our proof-of-concept study deliberately uses only one well-characterized batch of barley, thereby minimizing biological variability and allowing us to focus exclusively on the PEF effect. The study results thus represent a pivotal step toward advancing knowledge in this field.

## 4. Materials and Methods

### 4.1. Chemicals

Methanol, isopropanol, ammonium formate, ammonium acetate, and formic acid (all LC-MS-grade) were obtained from Merck (Darmstadt, Germany). Deionized water (18 MΩ, 25 °C) was prepared using a Milli-Q^®^ system (Millipore, Bedford, MA, USA).

### 4.2. Treated Barley Material

For the PEF experiment and the subsequent malting procedure, the barley cultivar Bojos—commonly used in the production of Pilsner-type malt—was selected. The barley, cultivated in the trial fields of the Crop Research Institute in Prague, was harvested in 2022. The identical material was previously employed in the study by Stranska et al. [70], which provides detailed data on its natural contamination with three *Fusarium* species (*F. graminearum*, *F. poae*, and *F. sporotrichioides*). In brief, the material contained 0.2423 ± 0.1524 ng/g of *F. graminearum*, 0.0014 ± 0.0011 ng/g of *F. sporotrichioides*, and 1.3307 ± 0.2613 ng/g of *F. poae*. For the subsequent PEF treatment and malting experiments, a one-kilogram batch of the harvested barley was prepared.

### 4.3. PEF Treatment of Malting Barley

The PEF treatment was carried out following the procedure described by Prusova et al. [60]. The PEF system (OMNIPEF, VITAVE, Czech Republic), equipped with a cylindrical batch chamber of 7 cm internal diameter, was employed to process one kilogram of barley. Prior to treatment, the barley grains were soaked for 10 min in a 0.05 M phosphate buffer solution to enhance current conductivity. The procedure was conducted under the following conditions: a voltage of 6 kV/cm, a current of 100 A, 100 bipolar pulses (both positive and negative), and a pulse width of 20 µs. The total specific energy delivered to the barley reached 152 J/g, calculated as (voltage × current × treatment time × number of pulses) divided by the sample weight, as reported by Prusova et al. [60]. In parallel, one kilogram of control barley (not subjected to PEF treatment) was soaked in the same phosphate buffer solution for 10 min and then immediately used for the malting process.

### 4.4. Malting Experiment Conditions

**Figure 4:**
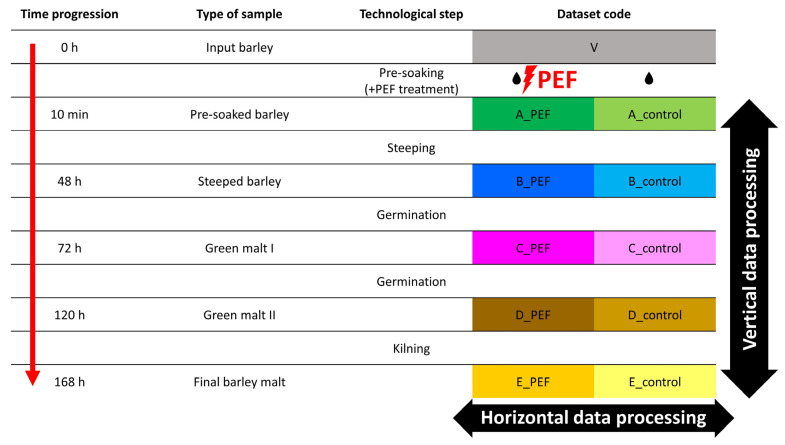
The schematic of samples collected during malting that represents the whole dataset. Two different data processing approaches, *Horizontal* and *Vertical*, are illustrated with black arrows.

The malting experiment was performed following the procedure described by Prusova et al. [60]. An automatic micro-malting system (RAVOZ^®^, Czech Republic), consisting of three separate units for steeping, germination, and kilning, was used. One kilogram of both PEF-treated and control barley was subjected to malting. The Pilsen-type malt was obtained under the following conditions: steeping for 48 h at 15 °C (8 h steeping; 12 h aeration rest; 8 h steeping; 12 h aeration rest; 4 h steeping; 4 h dripping), germination for 72 h at 15 °C with 95–98% humidity, and kilning for 24 h with a drying air temperature gradient from 45 °C to 82 °C (45 °C for 6 h; 50 °C for 6 h; gradual heating from 50 °C to 82 °C over 10 h; and 82 °C for 2 h). Approximately 100 g of input barley and samples from each technological stage of malting, for both the control and PEF-treated batches, were frozen at –80 °C for subsequent extraction. The complete set of samples from both malting experiments is presented in Figure 4.

### 4.5. Preparation and Extraction of Malting Samples for Metabolomic Fingerprinting

All frozen samples (in ten biological replicates) were homogenized using a cryogenic laboratory mill (IKA A11 basic, IKA Works GmbH & Co. KG, Staufen, Germany). The dry matter was determined for each type of sample (technological steps of malting) using a halogen moisture analyzer (Mettler Toledo HG63, Mettler-Toledo, Columbus, OH, USA). 1 g of dry matter was weighed into the 50 mL PTFE cuvette, and the corrected amount of methanol (9.18 mL) was added. The most dried samples were fortified with tap water to finally obtain 10 mL of extraction solvent methanol/water (9.18:0.82, *v*/*v*). Extraction solvent addition and sample weights are summarized in Table S3. This correction eliminated possible differentiation of samples due to the different water content of each technological step of malting. The cuvette with the homogenized sample was then placed on a laboratory shaker (HS 260 basic, IKA Works GmbH & Co. KG, Staufen, Germany) for 30 min (shaking at 240 RPM). The extract was then centrifuged (Rotina 35 R, Hettich Zentrifugen, Tuttingen, Germany) for 2 min at 13,528 g and microfiltered through 0.2 µm spin filters (Ciro, Deerfield Beach, FL, USA). Finally, an aliquot of approx. 1 mL was transferred to a glass HPLC vial for further analyses by UHPLC-HRMS/MS. To eliminate potential system drift in the analytical system, the pooled extract (quality control sample (QC)) was prepared by mixing 10 µL of extract from each sample and analyzed. To exclude background signals from laboratory processing of samples, a “processing blank” sample was prepared together with the analyzed samples.

### 4.6. The UHPLC-HRMS/MS Metabolomic Fingerprinting

UHPLC-HRMS/MS analysis was carried out in accordance with the Behner et al. [24]. For the separation of metabolites, the UHPLC system (Dionex UltiMate 3000 RS UHPLC system; Thermo Fisher Scientific; Waltham, MA, USA) used a reverse phase column of Acquity UPLC^®^ BEHC18 (100 mm × 2.1 mm; 1.7 m; Waters, United States). UHPLC method details are summarized in Table 3.

The SCIEX TripleTOF^®^ 6600 quadruple time-of-flight mass spectrometer (SCIEX, Concord, ON, Canada) was used for the detection in ESI- and ESI+ ionization modes. Ion source parameters are summarized in Table 4.

The calibration delivery system (CDS) was used to perform automatic *m*/*z* calibration of the MS system for up to ten consecutive samples, employing either negative or positive APCI calibration solutions (SCIEX, Concord, ON, Canada). The resolving power exceeded 40,000 FWHM, with reference masses of *m*/*z* 829.5393 (ESI+) and *m*/*z* 933.6370 (ESI−). To record MS1 and MS/MS data, the TOF MS method (full scan) and the Information Dependent Acquisition (IDA) method were applied. The operational *m*/*z* range was 100–1200 for MS1 and 50–1000 for MS/MS, with data collection carried out between 0.5 and 19 min. MS/MS spectra were acquired from the eight most intense precursor ions of the MS spectra. The collision energy was set to 35 ± 15 V, and the QC sample was analyzed every ten samples in the sequence. Instrument control and data acquisition were conducted using Analyst 1.7.1 TF software (SCIEX, Concord, ON, Canada), while qualitative data analysis was performed with SCIEX OS software (v. 1.5.0.23389, SCIEX, Concord, ON, Canada).

### 4.7. Data Processing

For data processing and analysis, the freely available MS-DIAL—MS-CleanR—MS-FINDER software platform was employed, following the workflow described by Behner et al. [24]. The UHPLC-HRMS/MS data were initially processed using MS-DIAL (v. 4.80, 2021, RIKEN, Japan). For peak picking, MS1 and MS/MS mass tolerances were set to 0.03 and 0.1 Da, respectively, in centroid mode for both ESI- and ESI+ datasets. A QC reference file was utilized for peak alignment, applying a retention time (RT) tolerance of 0.05 min and a mass tolerance of 0.015 Da. The minimum peak height threshold for detection was defined as an amplitude of 7000. During export, zero values in the MS-DIAL aligned results were replaced with one-tenth of the minimum peak height across all datasets. The aligned features were subsequently cleaned using the MS-CleanR (MetaToul-AgromiX Platform) tool. Filters for blanks, ghost peaks, incorrect masses, relative standard deviation (RSD), and relative mass defect (RMD) were applied, with a minimum blank ratio of 0.8, maximum RSD of 30%, and RMD values ranging from 50 to 3000. For Pearson correlation and ESI+/− data merging, the maximum RT and mass difference tolerances were set to 0.025 min and 0.005 Da, respectively. Feature clustering was carried out using Pearson correlation and the MS-DIAL peak character estimation (MS-DIAL-PCE) algorithm, retaining the most intense and most connected peak within each cluster. The filtered features were then automatically annotated in MS-FINDER (v. 3.52, 2021, RIKEN, Japan), with tolerances of 5 ppm for MS1 and 15 ppm for MS/MS. The formula finder included the elements C, H, O, N, P, and S. For compound identification, the following databases were queried: YMDB, ECMDB, PlantCyc, ChEBI, T3DB, NPA, KNApSAcK, LipidMaps, and PubChem. MS-FINDER provided multiple candidate chemical formulas and associated structures, ranked by score for each parameter. In the final step, MS-CleanR matched features from ESI+ and ESI- ionization modes, selected the top-ranked MS-FINDER annotations, and generated the final data matrix for subsequent statistical analysis. The complete dataset (see Figure 4) was processed using both *Horizontal* and *Vertical* approaches.

### 4.8. Statistical Analysis

In the case of the *Horizontal* data processing approach, the data matrix comprising two groups (PEF-treated/control) representing each technological step of malting (A, B, C, D, E) was filtered using univariate tools (Volcano plot—*t*-test, fold change) to prevent overfitting of the final models and to exclude statistically insignificant features. Univariate analysis was performed using the freely available web platform MetaboAnalyst (v. 5.0, Xia Lab, McGill, Canada), with a *t*-test false discovery rate (FDR) adjusted *p*-value < 0.01 and fold change >2. For the *Vertical* data processing approach, each data matrix representing the PEF-treated and control batches of the malting process (except dataset E—final malt, which was influenced by thermal treatment) was filtered using ANOVA with an FDR-adjusted *p*-value < 0.01. An overview of feature reduction during data filtration for all data matrices is summarized in Table S4. The final filtered and annotated data matrices from the *Horizontal* and *Vertical* approach, containing only the annotated features, were processed in MS Excel to perform normalization of the data (total area sum normalization). The normalized data were then uploaded to SIMCA software (v. 17.0, 2021, Umetrics, Sweden), where multivariate Principal Component Analysis (PCA) was applied. For the *Horizontal* approach, performed with slight modification according to Behner et al., 2025 [24], Orthogonal Partial Least Squares Discriminant Analysis (OPLS-DA) was subsequently conducted for each technological step of malting (A, B, C, D, E). Before building each classification model, Pareto scaling and logarithmic transformation were applied to the data. The quality of the models was evaluated using the R^2^Y and Q^2^ validation parameters, calculated via a seven-fold internal cross-validation, and a misclassification table (MT), which summarizes how accurately the models classify the samples into the known classes. Candidate biomarker compounds were selected based on OPLS-DA S-plots and VIP plots in combination with ROC. The simultaneous application of these filters ensured the selection of only statistically significant and relevant biomarkers. VIP scores greater than one (VIP score > 1) and a ROC area under the curve (AUC) equal to 1 were used as criteria. The biomarker selection process was verified using boxplots illustrating the representation of each biomarker in the tested groups (PEF-treated/control). In the *Vertical* approach, the Pattern Hunter algorithm (MetaboAnalyst v. 5.0, Xia Lab, McGill, Canada) was used to monitor changes during malting across the time series for the PEF-treated and control barley batches. Predefined increasing (1-2-3-4) and decreasing (4-3-2-1) patterns (Spearman’s rank correlation coefficient > 0.95) were used to select biomarkers showing clear increasing or decreasing trends throughout malting. The selection process was confirmed by boxplots showing the distribution of each biomarker across the examined groups (PEF-treated: A, B, C, D/control: A, B, C, D).

## 5. Conclusions

To the best of our knowledge, this is the first study describing the direct impact of PEF treatment on the malting process of barley. Based on our fingerprinting results, significant differences in the metabolome were observed between PEF-treated and control samples at each technological step of malting. A focus on metabolomic differences at each technological step revealed numerous down- or up-regulated biomarkers associated with PEF treatment. Most biomarkers, including 1,2-diacylglycerol-3-phosphates, triacylglycerols, linoleic acids and their derivatives, octadecanoids, N-acylserotonins, and very long-chain fatty acids, were up-regulated, likely reflecting the plant’s response to the abiotic stress induced by PEF treatment. The observed changes in metabolic profiles throughout the malting process indirectly indicated a possible enhancement of germination induced by PEF treatment. The hypothesis that PEF treatment may exhibit increased lipolytic and proteolytic activity in PEF-treated samples was further supported by the observation of elevated levels of metabolites from the following chemical groups: linoleic acids and derivatives, fatty acid esters, long-chain ceramides/fatty acids and alcohols, and the N-acylamines.

Although the overall pattern of the malting process in PEF-treated samples remained comparable to that of conventional untreated malt, advanced UHPLC-HRMS/MS analysis nevertheless detected significant metabolomic differences between treated and control samples. It is evident from the findings of this study that PEF treatment induces a specific form of abiotic stress in barley. However, these effects do not have a negative impact on the technological parameters of the final malt, and in terms of mold and mycotoxin mitigation, PEF appears to exert predominantly beneficial effects.

## Figures and Tables

**Table 1:** List of the most frequently occurring unique ontologies represented by two or more biomarker candidates related to PEF treatment (UP/DOWN-regulation) for each technological phase of malting.

Dataset	Type of Sample	PEF-Related Trend	Unique Ontology *
A	Pre-soaked barley	↑ UP-regulated	1,2-diacylglycerol-3-phosphates
B	Steeped barley	↑ UP-regulated	Lineolic acids and derivatives; Triacylglycerols; Octadecanoids; Diterpenoids
B	Steeped barley	↓ DOWN-regulated	1-(1Z-alkenyl),2-acylglycerophosphoinositols; Furanoid fatty acids; Hybrid peptides; N-acylserotonins
C	Green malt I	↑ UP-regulated	N-acyl-alpha amino acids and derivatives; Beta carbolines, N-acylserotonins, Triacylglycerols
D	Green malt II	↓ DOWN-regulated	Sesquiterpenoids; Linoleic acids and derivatives; 1,2-diacylglycerols; Fatty acid esters; Fatty alcohols; Prostaglandins and related compounds
D	Green malt II	↑ UP-regulated	Triacylglycerols; Very long-chain fatty acids
E	Final barley malt	↓ DOWN-regulated	Acyclic terpenoids
E	Final barley malt	↑ UP-regulated	Very long-chain fatty acids

* Unique chemical group of biomarkers represented by two or more candidates for a specific group of datasets, sorted by occurrence—the first ontology is the most frequently occurring in the specific group; for datasets A (pre-soaked barley) and C (green malt I), no unique ontologies containing two or more biomarker candidates showing down-regulation following PEF treatment were identified.

**Table 2:** List of the most frequently occurring unique ontologies represented by two or more biomarker candidates with increasing (1-2-3-4) or decreasing (4-3-2-1) trend in the ‘A-B-C-D’ direction for PEF malting and control malting experiments.

Dataset	Trend	Unique Ontology *
PEF	↑ 1-2-3-4	Triterpenoids, Linoleic acids and derivatives, 2-arylbenzofuran flavonoids, Diterpene glycosides, Fatty acid esters, Glycosphingolipids, N-acyl amines, Xanthophylls, Long-chain ceramides/fatty acids/fatty alcohols
PEF	↓ 4-3-2-1	1,2-diacylglycerol-3-phosphates, Triacylglycerols, Phosphocholines
control	↑ 1-2-3-4	Triterpenoids
control	↓ 4-3-2-1	1,2-diacylglycerol-3-phosphates, 1-acyl-sn-glycero-3-phosphocholines, Fatty alcohols, Long-chain fatty acids

* Unique chemical group of biomarkers represented by two or more candidates for a specific group of datasets, sorted by occurrence—the first ontology is the most frequently occurring in the specific group.

**Table 3:** UHPLC method details.

injection volume	2 µL
autosampler temperature	10 °C
column temperature	60 °C
mobile phase composition	(A) 5 mM ammonium in milli-Q water/methanol (95:5, *v*/*v*) with 0.1% formic acid (*v*/*v*) (B) 5 mM ammonium in isopropanol/methanol/milli-Q water (65:30:5, *v*/*v*/*v*) with 0.1% formic acid (*v*/*v*)
elution gradient	0.0 min (10% B; 0.4 mL/min) 1.0 min (50% B; 0.4 mL/min) 5.0 min (80% B; 0.4 mL/min) 11.0 min (100% B; 0.4 mL/min) 19.0 min (100% B; 0.4 mL/min) 19.01 min (10% B; 0.4 mL/min) 21.0 min (10% B; 0.4 mL/min)

**Table 4:** Settings of the ion source parameters.

curtain gas pressure	35 psi
nebulizing gas pressure	55 psi
drying gas pressure	55 psi
temperature	500 °C
capillary voltage	−4.5 kV (ESI-)/+4.5 kV (ESI+)
declustering potential	80 V

## Data Availability

The data presented in this study are available in Supplementary Materials, https://www.mdpi.com/1420-3049/30/4/924#app1-molecules-30-00924 (accessed on 30 September 2025).

## Supplementary Materials

The following supporting information can be downloaded at: https://www.mdpi.com/article/10.3390/molecules30193953/s1. Table S1: Number of metabolomic features according to their polarity gained from UHPLC-HRMS records of various extraction solvents and their mixtures; Table S2: Quality parameters (R2Y, Q2) and Misclassification table results (MT) of OPLS-DA models; Table S3: Extraction solvent addition and sample weights; Table S4: The general overview of the features reduction during data filtration for all data matrices; Figures S1–S5: UHPLC-HRMS fingerprints of PEF treated (green) and control (blue) samples; MeOH extracts, ESI+. The zoomed areas of the TIC chromatograms highlight the differences in the low-intensity regions; Figure S6: Graph illustrating the ratio of statistically significant variables with good peak shape and statistically significant variables discarded for five tested thresholds of the Minimum peak height processing parameter in MS-DIAL software; Figure S7: The OPLS-DA models (Score scatter plot) of each individual technological steps of malting (A—Pre-soaked barley, B—Steeped barley, C—Green malt I, D—Green malt II; Figure S8: Heatmap visualization of increasing (1-2-3-4) or decreasing (4-3-2-1) patterns of ontologies represented by specific biomarkers selected by Pattern Hunter (Spearman’s rank correlation coefficient > 0.95) for control malting experiment; Figure S9: Heatmap visualization of increasing (1-2-3-4) or decreasing (4-3-2-1) patterns of ontologies represented by specific biomarkers selected by Pattern Hunter (Spearman’s rank correlation coefficient > 0.95) for PEF malting experiment. Excel files: Candidates of PEF-related biomarkers Horizontal and Candidates of PEF-related biomarkers Vertical.
