# Supplementary material for: Pulsed Electric Fields Reshape the Malting Barley Metabolome: Insights from UHPLC-HRMS/MS
Source: Molecules. 2025 Oct 1;30(19):3953. doi: 10.3390/molecules30193953 (PMC12525893; doi:10.3390/molecules30193953)
Supplement: Supplementary file 1 [file molecules-30-03953-s001.zip › Supplementary_Behner_barley-metabolomics_Molecules.pdf]

Supplementary material to:

Pulsed Electric Fields Reshape the Malting Barley Metabolome: Insights from UHPLC-HRMS/MS

Adam Behner [1], Nela Prusova [1], Marcel Karabin [2], Lukas Jelinek [2], Jana Hajslova [1] and Milena Stranska [1,*]

[1] Department of Food Analysis and Nutrition, University of Chemistry and Technology, Technicka 3, 166 28 Prague, Czech Republic; adam.behner@vscht.cz (A.B.); nela.prusova@vscht.cz (N.P.); hajslovj@vscht.cz (J.H.)

[2] Department of Biotechnology, University of Chemistry and Technology, Prague, Technicka 5, 166 28 Prague, Czech Republic; marcel.karabin@vscht.cz (M.K.); lukas.jelinek@vscht.cz (L.J.)

* Correspondence: milena.stranska@vscht.cz; Tel. +420-220-443-142

Table S1: Number of metabolomic features according to their polarity gained from UHPLC-HRMS/MS records of various extraction solvents and their mixtures.

Polarity of features	Extraction solvent / solvent mixtures	Number of features		
		ESI+	ESI-	SUM.
Polar (0-6 min Rt)	water	2725	1812	4537
	methanol:water (50:50, v/v)	3688	2546	6234
	**methanol**	**3370**	**2120**	**5490**
	methanol:propan-2-ol (50:50, v/v)	2512	1590	4102
Middle-polar (6-12 min Rt)	water	310	106	416
	methanol:water (50:50, v/v)	459	186	645
	**methanol**	**4641**	**2583**	**7224**
	methanol:propan-2-ol (50:50, v/v)	4738	2337	7075
Nonpolar (12-19 min Rt)	water	730	12	742
	methanol:water (50:50, v/v)	727	6	733
	**methanol**	**3094**	**739**	**3833**
	methanol:propan-2-ol (50:50, v/v)	3542	710	4252

Table S2: Quality parameters (R^2Y, Q^2) and Misclassification table results (MT) of OPLS-DA models.

Dataset	R^2Y	Q^2	Correct percentage (MT) PEF/control
A	0.995	0.990	100% / 100%
B	0.997	0.990	100% / 100%
C	0.999	0.997	100% / 100%
D	0.998	0.990	100% / 100%
E	0.998	0.996	100% / 100%

Table S3: Extraction solvent addition and sample weights

Type of sample	Dataset code	Approx. dry matter content (%)	Approx. water content (%)	Corrected weight [g]	Natural water presence [g]	Water addition [g]	MeOH [mL]	Total solvents [ml]	Matrix / solvent ratio
Input barley	V	90.5	9.5	1.10	0.10	0.71	9.18	10.00	0.1
Pre-soaked barley	A	74	26	1.35	0.35	0.47	9.18	10.00	0.1
Steeped barley	B	55	45	1.82	0.82	0.00	9.18	10.00	0.1
Green malt I	C	55	45	1.82	0.82	0.00	9.18	10.00	0.1
Green malt II	D	55	45	1.82	0.82	0.00	9.18	10.00	0.1
Final barley malt	E	95	5	1.05	0.05	0.77	9.18	10.00	0.1

Table S4: The general overview of the features reduction during data filtration for all datasets

	Number of features				
Data treatment step	Processing	Filtration	Automatic identification	Statistical filter	
Software tool	MS-DIAL	MS-CleanR	MS-FINDER	Metaboanalyst: t-test (p-value <0.01, FDR)	Metaboanalyst: ANOVA (p-value <0.01, FDR)
A (ESI+/-)	19,265	981	173	83	–
B (ESI+/-)	19,756	883	494	256	–
C (ESI+/-)	19,521	965	544	245	–
D (ESI+/-)	22,291	1115	580	379	–
E (ESI+/-)	22,674	1042	558	254	–
Vertical_PEF	26,984	1631	859	–	853
Vertical_control	27,051	1634	854	–	845

Figure S1: UHPLC-HRMS/MS fingerprints represented by raw-data TIC of PEF treated (green) and control (blue) Pre-soaked barley (dataset **A**) samples; MeOH extracts, ESI+. The zoomed areas of the TIC chromatograms highlight the differences in the low-intensity regions.

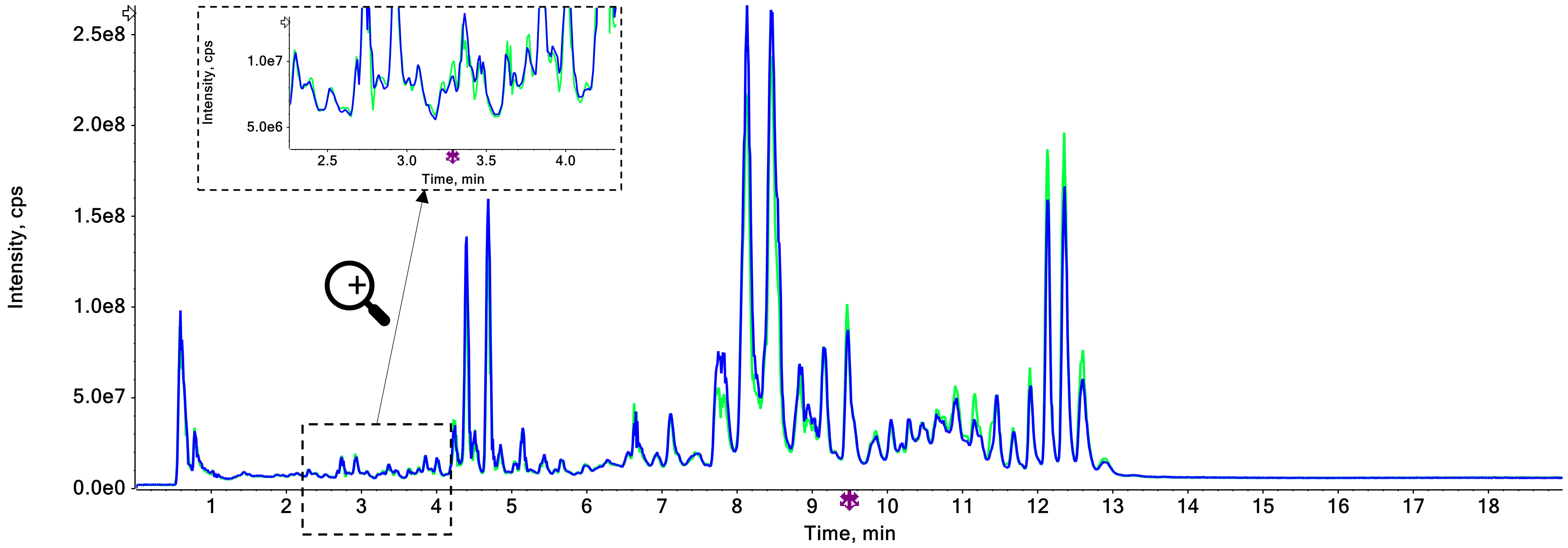

Figure S2: UHPLC-HRMS/MS fingerprints represented by raw-data TIC of PEF treated (green) and control (blue) Steeped barley (dataset **B**) samples; MeOH extracts, ESI+. The zoomed areas of the TIC chromatograms highlight the differences in the low-intensity regions.

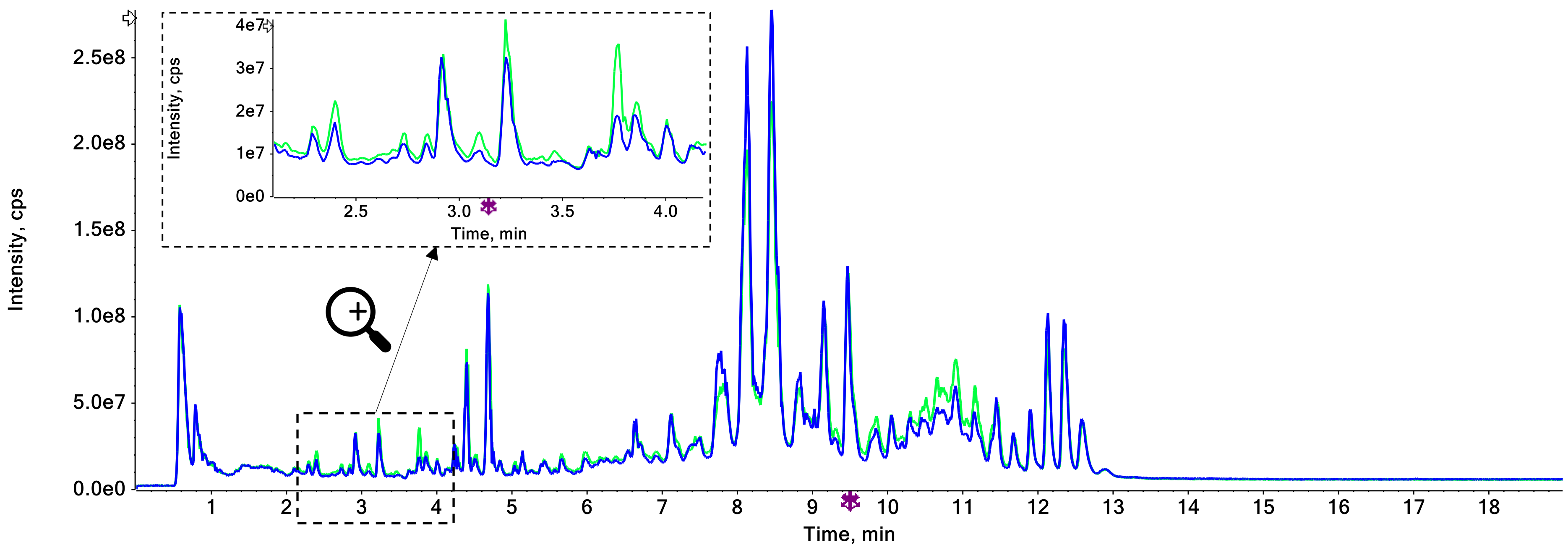

Figure S3: UHPLC-HRMS/MS fingerprints represented by raw-data TIC of PEF treated (green) and control (blue) Green malt I (dataset **C**) samples; MeOH extracts, ESI+. The zoomed areas of the TIC chromatograms highlight the differences in the low-intensity regions.

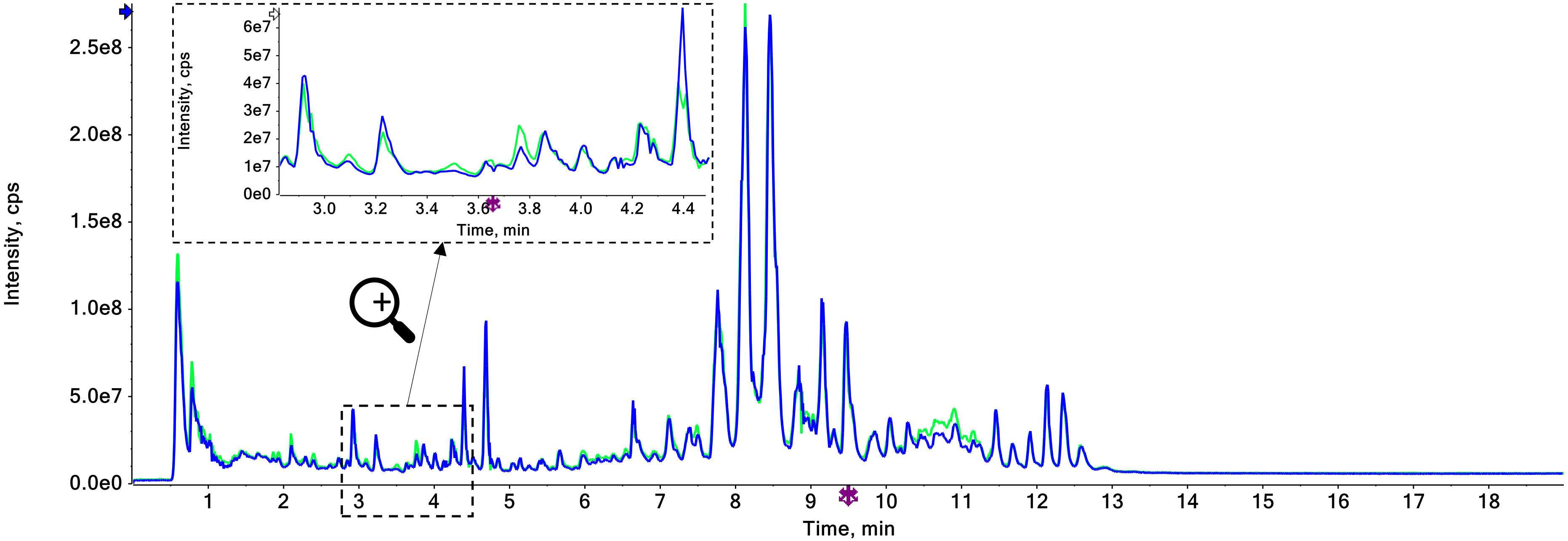

Figure S4: UHPLC-HRMS/MS fingerprints represented by raw-data TIC of PEF treated (green) and control (blue) Green malt II (dataset **D**) samples; MeOH extracts, ESI+. The zoomed areas of the TIC chromatograms highlight the differences in the low-intensity regions.

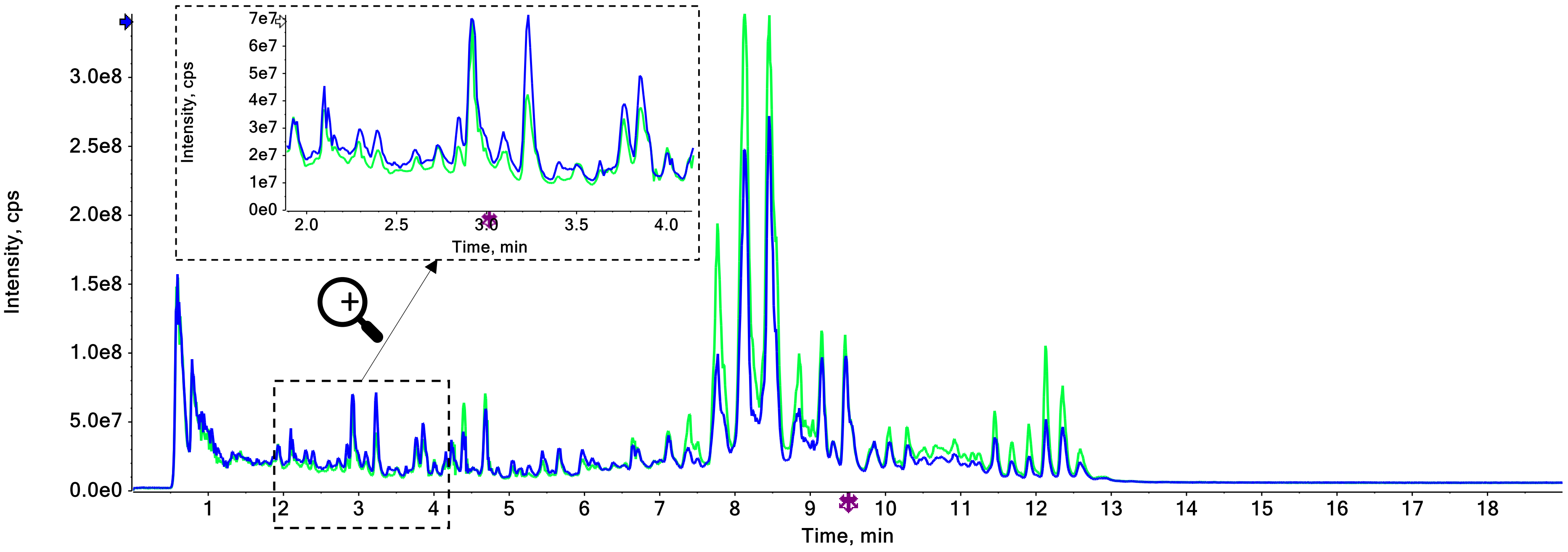

Figure S5: UHPLC-HRMS/MS fingerprints represented by raw-data TIC of PEF treated (green) and control (blue) Final barley malt (dataset **E**) samples; MeOH extracts, ESI+. The zoomed areas of the TIC chromatograms highlight the differences in the low-intensity regions.

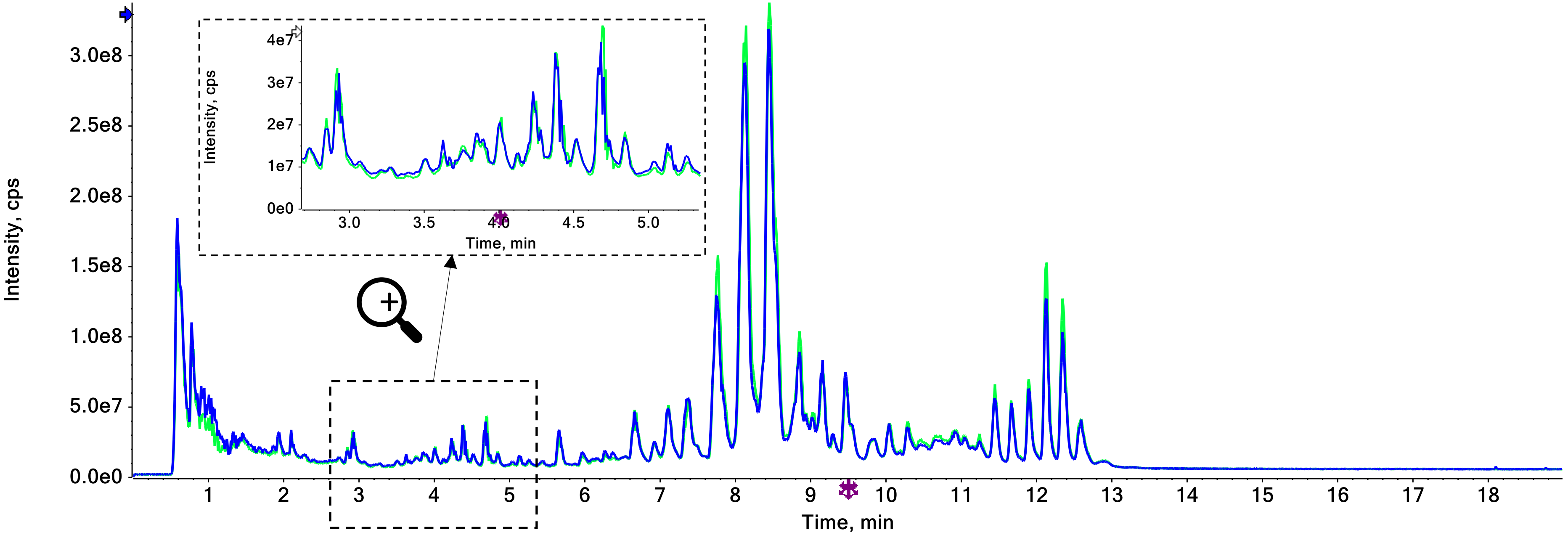

Figure S6: Graph illustrating the ratio of statistically significant variables with good peak shape and statistically significant variables discarded for five tested thresholds of the *Minimum peak height* processing parameter in MS-DIAL software.

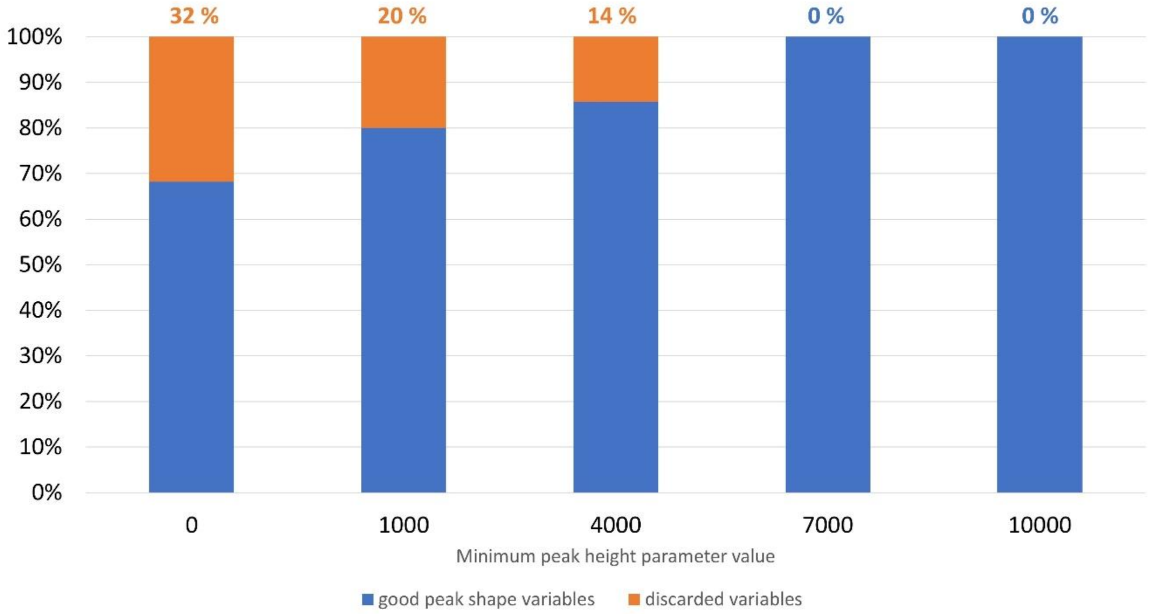

Figure S7: The OPLS-DA models (Score scatter plot) of each individual technological steps of malting (n=10; **A** – Pre-soaked barle**y, B** – Steeped barley, **C** – Green malt I, **D** – Green malt II, **E** – Final barley malt) colored according to PEF-treatment (PEF) and control with excellent values of quality parameters.

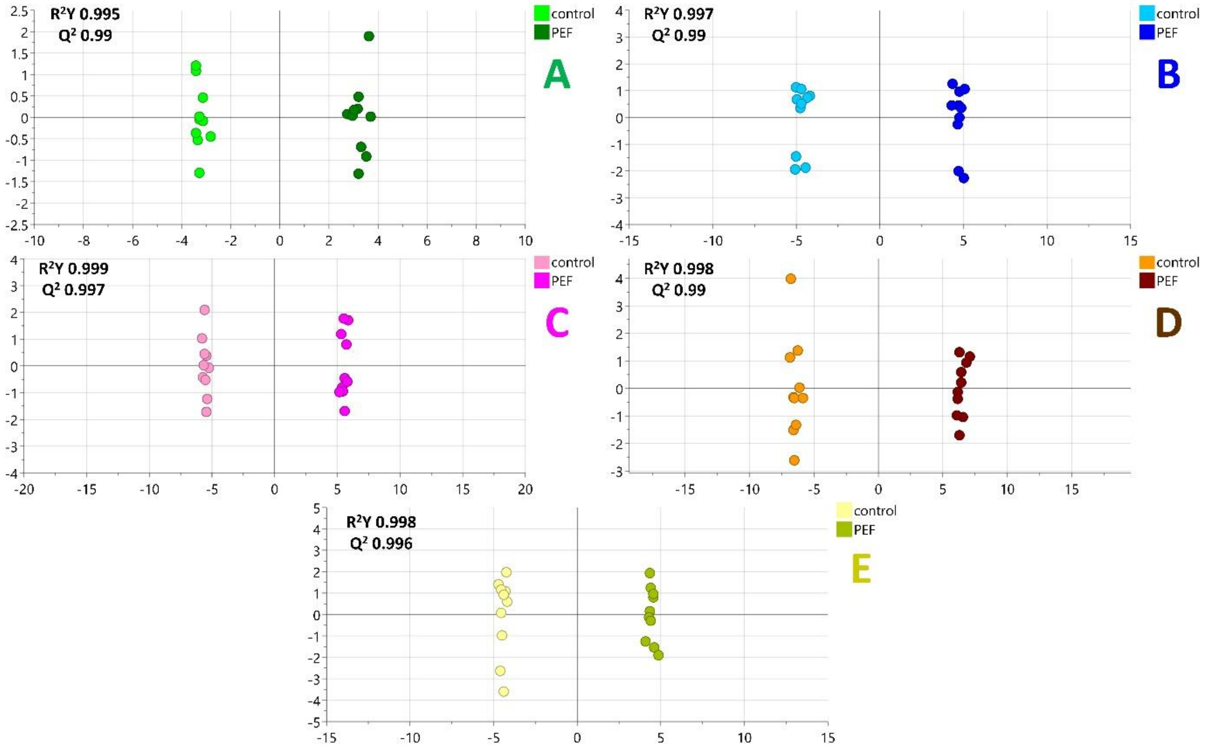

Figure S8: Heatmap visualization of increasing (1-2-3-4) or decreasing (4-3-2-1) trend ('A-B-C-D' direction of malting process) of ontologies represented by specific biomarkers selected by Pattern Hunter (Spearman's rank correlation coefficient > 0.95) for control malting experiment. Lower intensities of biomarkers are visualized with yellow color and higher intensities with purple color. To highlight the unique differences between PEF-treated and control samples, ontologies exhibiting both trends (1-2-3-4 and 4-3-2-1) within the same dataset group (PEF or control) were excluded from the heatmap construction. Labels of rows representing abbreviations of ontologies (see the legend under heatmap) with specific feature IDs of represented biomarker. The vertical axis represents technological steps of control-malting experiment (green A – pre-soaked and treated barley, blue B – steeped barley, pink C – green malt I, orange D – green malt II). All biomarkers with detail information are listed in the supplementary file **Candidates of PEF-related biomarkers_Vertical.xlsx**.

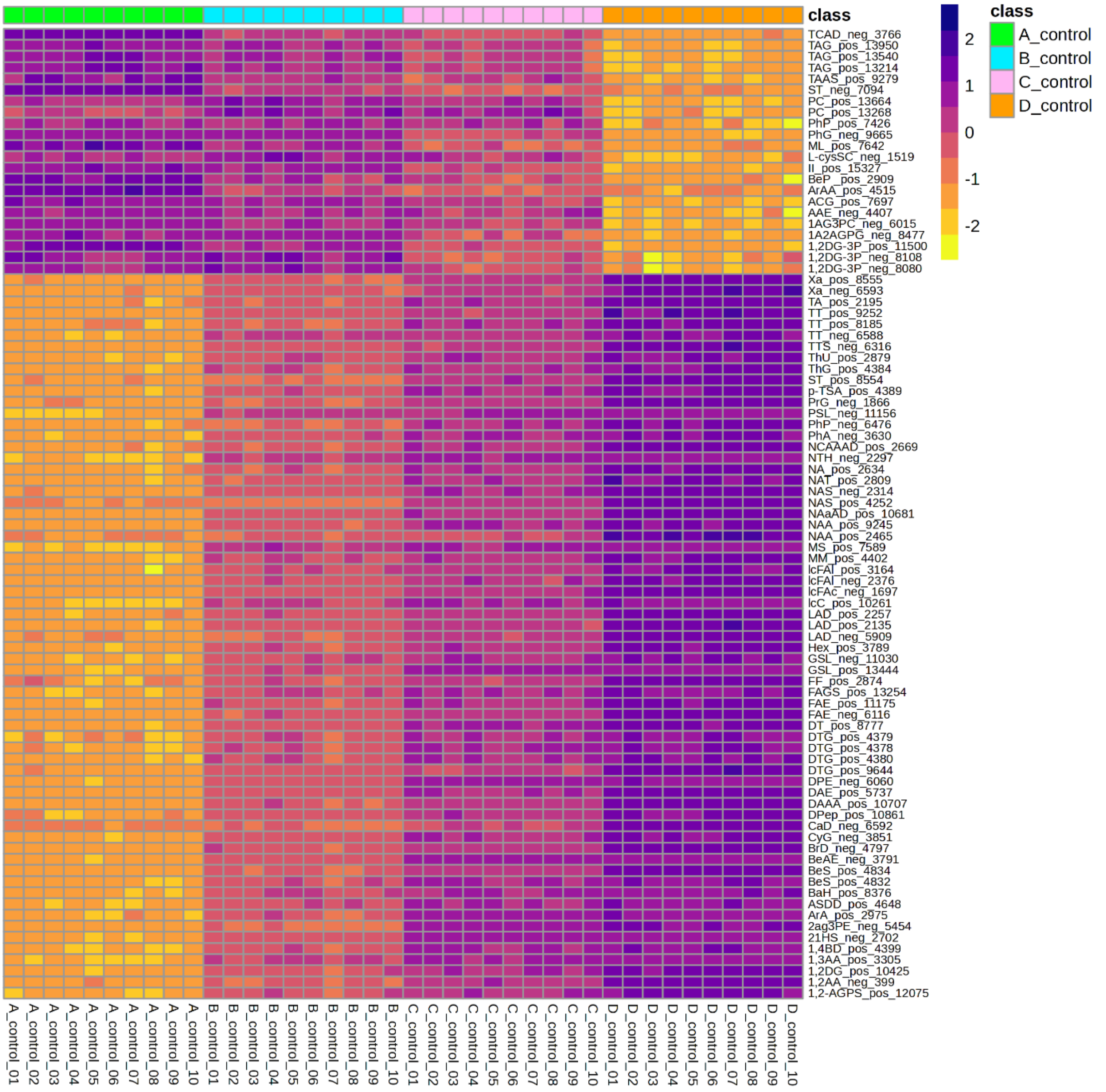

Legend:

**Abbreviation Ontology**	**Ontology**
TCAD	Tricarboxylic acids and derivatives
TAG	Triacylglycerols
TAG	Triacylglycerols
TAG	Triacylglycerols
TAAS	Tetraalkylammonium salts
ST	Sesterterpenoids
PC	Phosphocholines
PC	Phosphocholines
PhP	Phenylpyrimidines
PhG	Phenolic glycosides
ML	Macrolactams
L-cysSC	L-cysteine-S-conjugates
II	Isoindolones
BeP	Benzopyrenes
ArAA	Aralkylamines
ACG	Aminocyclitol glycosides
AAE	Alkyl aryl ethers
1AG3PC	1-acyl-sn-glycero-3-phosphocholines
1A2AGPG	1-acyl,2-alkylglycerophosphoglycerols
1,2DG-3P	1,2-diacylglycerol-3-phosphates
1,2DG-3P	1,2-diacylglycerol-3-phosphates
1,2DG-3P	1,2-diacylglycerol-3-phosphates
Xa	Xanthophylls
Xa	Xanthophylls
TA	Tropane alkaloids
TT	Triterpenoids
TT	Triterpenoids
TT	Triterpenoids
TTS	Triterpene saponins
ThU	Thioureas
ThG	Thioglycosides
ST	Sesquaterpenoids
p-TSA	P-toluenesulfonamides
PrG	Prostaglandins and related compounds
PSL	Phosphosphingolipids
PhP	Phenylpyridines
PhA	Phenylazetidines
NCAAAD	N-carbamoyl-alpha amino acids and derivatives
NTH	Naphthyridines
NA	Naphthalenes
NAT	N-aliphatic s-triazines
NAS	N-acylserotonins
NAS	N-acylserotonins
NAaAD	N-acyl-alpha amino acids and derivatives
NAA	N-acyl amines
NAA	N-acyl amines
MS	Monosaccharides
MM	Menthane monoterpenoids
lcFAl	Long-chain fatty alcohols
lcFAl	Long-chain fatty alcohols
lcFAc	Long-chain fatty acids
lcC	Long-chain ceramides
LAD	Lineolic acids and derivatives
LAD	Lineolic acids and derivatives
LAD	Lineolic acids and derivatives
Hex	Hexoses
GSL	Glycosphingolipids
GSL	Glycosphingolipids
FF	Furofurans
FAGS	Fatty acyl glycosides of mono- and disaccharides
FAE	Fatty acid esters
FAE	Fatty acid esters
DT	Diterpenoids
DTG	Diterpene glycosides
DTG	Diterpene glycosides
DTG	Diterpene glycosides
DTG	Diterpene glycosides
DPE	Diphenylethers
DAE	Diarylethers
DAAA	Dialkylarylamines
DPep	Depsipeptides
CaD	Cycloartanols and derivatives

CyG	Cyanogenic glycosides
BrD	Brassinolides and derivatives
BeAE	Benzoic acid esters
BeS	Benzenesulfonyl compounds
BeS	Benzenesulfonyl compounds
BaH	Bacteriohopanoids
ASDD	Azaspirodecane derivatives
ArA	Aryl-aldehydes
2ag3PE	2-acyl-sn-glycero-3-phosphoethanolamines
21HS	21-hydroxysteroids
1,4BD	1,4benzodiazepines
1,3AA	1,3aminoalcohols
1,2DG	1,2diacylglycerols
1,2AA	1,2aminoalcohols
1,2-AGPS	1-(Z-alkenyl),2-acylglycerophosphoserines

Figure S9: Heatmap visualization of increasing (1-2-3-4) or decreasing (4-3-2-1) trend ('A-B-C-D' direction of malting process) of ontologies represented by specific biomarkers selected by Pattern Hunter (Spearman's rank correlation coefficient > 0.95) for PEF malting experiment. Lower intensities of biomarkers are visualized with yellow color and higher intensities with purple color. To highlight the unique differences between PEF-treated and control samples, ontologies exhibiting both trends (1-2-3-4 and 4-3-2-1) within the same dataset group (PEF or control) were excluded from the heatmap construction. Labels of rows representing abbreviations of ontologies (see the legend under heatmap) with specific feature IDs of represented biomarker. The vertical axis represents technological steps of PEF-malting experiment (green A – pre-soaked and treated barley, blue B – steeped barley, magenta C – green malt I, brown D – green malt II). All biomarkers with detail information are listed in the supplementary file **Candidates of PEF-related biomarkers_Vertical.xlsx**.

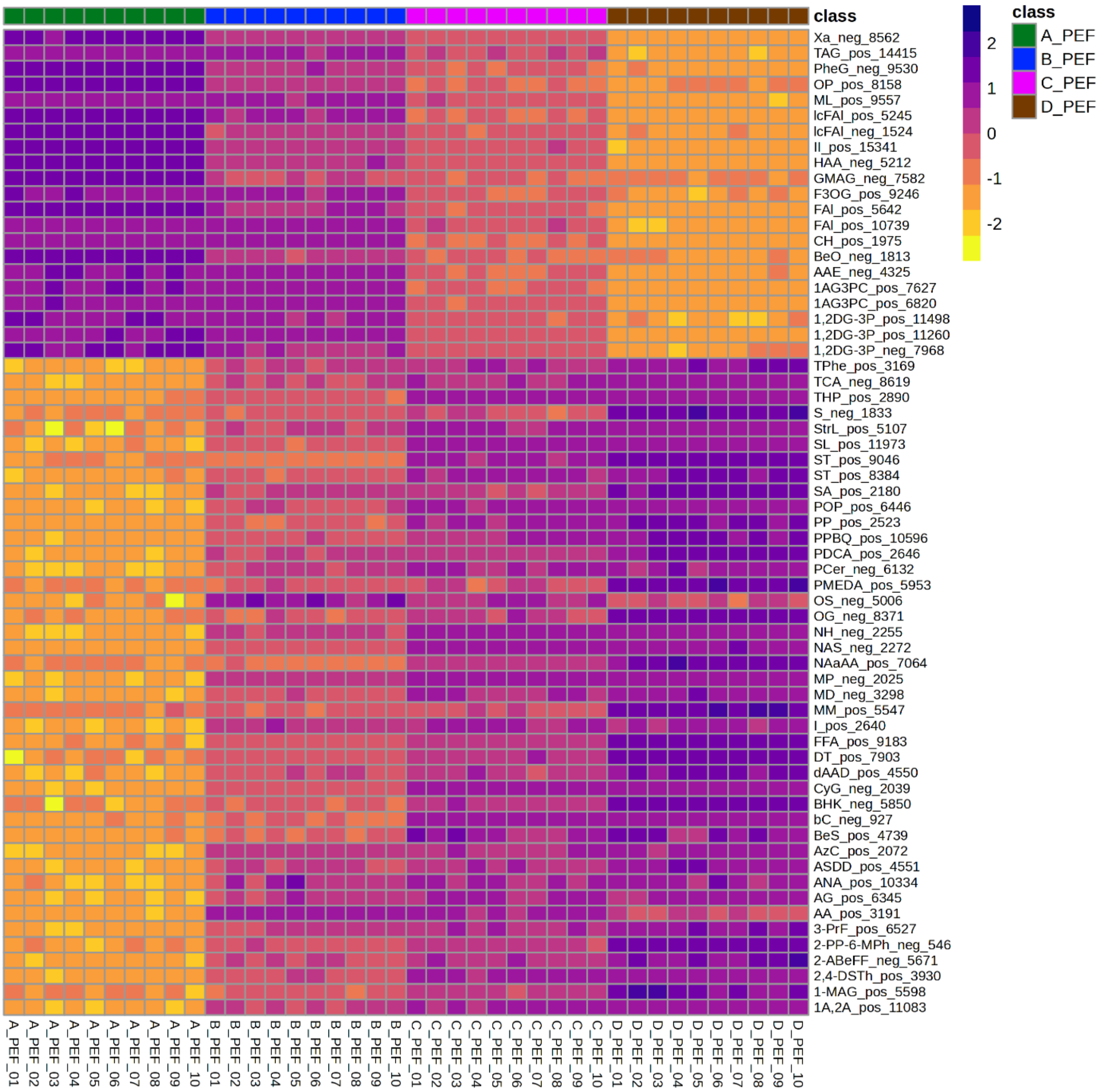

Legend:

Abbreviation Ontology	Ontology
Xa	Xanthophylls
TAG	Triacylglycerols

PheG	Phenolic glycosides
OP	Oligopeptides
ML	Macrolactams
lcFAl	Long-chain fatty alcohols
lcFAl	Long-chain fatty acids
II	Isoindolones
HAA	Hydroxamic acids
GMAG	Glycosylmonoacylglycerols
F3OG	Flavonoid-3-O-glycosides
FAl	Fatty alcohols
FAl	Fatty alcohols
CH	Cyclohexenones
BeO	Benzoxazoles
AAE	Alkyl aryl ethers
1AG3PC	1-acyl-sn-glycero-3-phosphocholines
1AG3PC	1-acyl-sn-glycero-3-phosphocholines
1,2DG-3P	1,2-diacylglycerol-3-phosphates
1,2DG-3P	1,2-diacylglycerol-3-phosphates
1,2DG-3P	1,2-diacylglycerol-3-phosphates
TPhe	Triphenyl compounds
TCA	Tricarboxylic acids and derivatives
THP	Tetrahydropyridines
S	Sulfones
StrL	Strychnos alkaloids
SL	Steroid lactones
ST	Sesquiterpenoids
ST	Sesquiterpenoids
SA	Secondary alcohols
POP	Pyrrolopyrimidines
PP	Pyrido[2,3-d]pyrimidines
PPBQ	Polyprenylbenzoquinones
PDCA	Piperidinecarboxamides
PCer	Phytoceramides
PMEDA	Phosphoric monoester diamides
OS	Oligosaccharides
OG	O-glycosyl compounds
NH	Naphthyridines
NAS	N-acylserotonins
NAaAA	N-acyl-alpha amino acids and derivatives
MP	Methoxypyrazines
MD	Methionine and derivatives
MM	Menthane monoterpenoids
I	Imidazolidines
FFA	Furanoid fatty acids
DT	Diterpenoids
dAAD	Delta amino acids and derivatives
CyG	Cyanogenic glycosides
BHK	Beta-hydroxy ketones
bC	Beta carbolines
BeS	Benzenesulfonyl compounds
AzC	Azoxy compounds
ASDD	Azaspirodecane derivatives
ANA	Annonaceous acetogenins
AG	Aminoglycosides
AA	Acetamides
3-PrF	3'-prenylated flavones
2-PP-6-MPh	2-polyprenyl-6-methoxyphenols
2-ABeFF	2-arylbenzofuran flavonoids
2,4-DSTh	2,4-disubstituted thiazoles
1-MAG	1-monoacylglycerols
1A,2A	1-alkyl,2-acylglycerophosphates
